# Fecal Microbiota Transplantation for Recurrent *Clostridioides difficile* Infections in a Cystic Fibrosis Child Previously Screen Positive, Inconclusive Diagnosis (CFSPID): A Case Report

**DOI:** 10.3390/microorganisms12102059

**Published:** 2024-10-12

**Authors:** Riccardo Marsiglia, Stefania Pane, Federica Del Chierico, Alessandra Russo, Pamela Vernocchi, Lorenza Romani, Sabrina Cardile, Antonella Diamanti, Luisa Galli, Agnese Tamborino, Vito Terlizzi, Paola De Angelis, Giulia Angelino, Lorenza Putignani

**Affiliations:** 1 Immunology, Rheumatology and Infectious Diseases Research Area, Unit of Research Microbiome, Bambino Gesù Children’s Hospital, IRCCS, 00146 Rome, Italy; riccardo.marsiglia@opbg.net (R.M.); federica.delchierico@opbg.net (F.D.C.); pamela.vernocchi@opbg.net (P.V.); 2 Unit of Microbiomics, Bambino Gesù Children’s Hospital, IRCCS, 00146 Rome, Italy; stefania.pane@opbg.net (S.P.); alessandra.russo@opbg.net (A.R.); 3 Infectious Diseases Unit, Bambino Gesù Children’s Hospital, IRCCS, 00146 Rome, Italy; lorenza.romani@opbg.net; 4 Unit of Gastroenterology and Nutrition, Bambino Gesù Children’s Hospital, IRCCS, 00165 Rome, Italy; sabrina.cardile@opbg.net (S.C.); antonella.diamanti@opbg.net (A.D.); paola.deangelis@opbg.net (P.D.A.); giulia.angelino@opbg.net (G.A.); 5 Department of Health Sciences, University of Florence, 50121 Florence, Italy; luisa.galli@unifi.it; 6 Infectious Disease Unit, Meyer Children’s Hospital IRCCS, 50121 Florence, Italy; agnese.tamborino@meyer.it; 7 Department of Pediatric Medicine, Meyer Children’s Hospital IRCCS, Cystic Fibrosis Regional Reference Center, Viale Gaetano Pieraccini 24, 50139 Florence, Italy; vito.terlizzi@meyer.it; 8 Unit of Microbiomics and Unit of Research of Microbiome, Bambino Gesù Children’s Hospital, IRCCS, 00146 Rome, Italy

**Keywords:** cystic fibrosis (CF), cystic fibrosis screen positive, inconclusive diagnosis (CFSPID), recurrent *Clostridioides difficile* infections (rCDIs), fecal microbiota transplantation (FMT), gut microbiota (GM)

## Abstract

*Clostridioides difficile* infection (CDI) is generally treated with vancomycin, metronidazole or fidaxomicin, although fecal microbiota transplantation (FMT) represents a promising therapeutic option for antibiotic-resistant recurrent *C. difficile* infections (rCDIs) in adults. In pediatric cystic fibrosis (CF) patients, CDIs are generally asymptomatic and respond to treatment. Here, we present the case of an 8-year-old female, initially diagnosed as “CFTR-related metabolic syndrome/cystic fibrosis screen positive, inconclusive diagnosis” (CMRS/CFSPID), who then progressed to CF at 12 months. In the absence of CF-related symptoms, she presented multiple and disabling episodes of bloody diarrhoea with positive tests for *C. difficile* antigen and A/B toxin. After conventional treatments failed and several CDI relapses, FMT was proposed. Donor screening and GM donor–receiver matching identified her mother as a donor. Metataxonomy and targeted metabolomics provided, through a pre- and post-FMT time course, gut microbiota (GM) profiling to assess GM engraftment. At first, the GM map revealed severe dysbiosis, with a prevalence of Bacteroidetes and Proteobacteria (i.e., *Klebsiella* spp., *Escherichia coli*), a reduction in Firmicutes, a GM nearly entirely composed of Enterococcaceae (i.e., *Enterococcus*) and an almost complete depletion of Verrucomicrobia and Actinobacteria, mostly represented by *Veillonella dispar*. Post FMT, an increment in *Bifidobacterium* spp. and *Collinsella* spp. with a decrease in *V. dispar* restored intestinal eubiosis. Consistently, four weeks after FMT treatment, the child’s gut symptoms cleared, without CDI recurrence.

## 1. Introduction

Cystic fibrosis (CF) is a multi-organ disease, primarily affecting the respiratory, digestive and reproductive systems and the sweat glands. The primary clinical manifestation of CF are exocrine pancreatic insufficiency and progressive pulmonary and gastrointestinal tract dysfunctions [1]. Moreover, patients with CF experience multiple bacterial infections throughout their lives [2,3]. Thus, antibiotic therapies in these patients are directed at preventing, eradicating or controlling respiratory infections [4]. However, these treatments also alter the gut microbiota (GM) composition and metabolic function, leading to a loss of commensal *Clostridioides* spp. and the onset of *Clostridioides difficile* infections (CDIs) [5]. *C. difficile* is a Gram-positive spore-forming bacterium. Its spores can spread by the fecal–oral pathway, pass the stomach barrier and germinate into vegetative forms [5], producing toxins that can damage the intestinal mucosa, causing various symptoms from inflammation, diarrhoea, fever, abdominal pain and nausea to severe complications, such as toxic megacolon and colonic perforation [6,7]. Normally, the GM protects the intestine against this pathogen by the production of short-chain fatty acids (SCFA) and secondary bile acids [8] and by stimulating the immune system [9]. The disruption of intestinal eubiosis may cause an overgrowth of the vegetative forms, leading to toxinogenic *C. difficile* colonization [10,11]. In this case, two different kinds of clostridial exotoxins are produced, specifically TcdA and TcdB, responsible for inflammation and intestinal damage [12]. After a first episode of CDI, a symptomatic recurrence, namely recurrent CDI (rCDI), can develop [13]. Spores are involved in the pathophysiology of rCDI, when the patient may become re-infected with the same or different *C. difficile* strains, causing a recurring inflammation [14], especially in the case of an inappropriate immune response [15].

In clinical practice, CDI is normally treated with antibiotic therapy, i.e., vancomycin, metronidazole or fidaxomicin [16]. However, fecal microbiota transplantation (FMT) is a therapeutic option for rCDIs in adults [17,18,19] and is still an experimental treatment in children, despite support from the American Society for Pediatric Gastroenterology, Hepatology, and Nutrition and the European Society for Pediatric Gastroenterology, Hepatology and Nutrition [20]. In the last few years, the use of FMT in children under different disease conditions has significantly increased and has progressively taken advantages from the growing knowledge on the ecology and function of the gut microbiota, including its degree of dysbiosis, as thoroughly reported in the literature (Supplementary Table S1).

FMT is the process of transplanting feces from healthy donors to recipients to introduce a healthy GM [21]. It can mitigate the negative effects of antibiotics by disrupting the receiver’s GM and restoring eubiosis [17]. FMT is considered a safe and affordable treatment to correct GM imbalance after CDI and restore normal bowel function [14].

## 2. Case Presentation Section

This is the case of an 8-year-old female followed at the Cystic Fibrosis Regional Reference Centre of Florence, Italy (Meyer Children’s Hospital IRCCS), initially diagnosed as CF transmembrane conductance regulator (CFTR)-related metabolic syndrome/cystic fibrosis screen positive, inconclusive diagnosis (CMRS/CFSPID) [22] in the presence of positive CF newborn screening, sweat chloride in the intermediate range (49–42 mmol/L) and *CFTR* genotype: F508del/S737F (less than two *CFTR*-causing variants) [23,24].

The patient then progressed to CF with pancreatic sufficiency at 12 months for pathological (63–68 mmol/L) sweat tests. During follow-up, no CF-related symptoms appeared, nor did lung disease or the need for antibiotic therapies.

The child presented multiple episodes of bloody diarrhoea between May 2022 and October 2023, with positive tests for *C. difficile* antigen and toxin A/B.

Different antibiotic therapies, including metronidazole, vancomycin and fidaxomicin, were administered, but only with temporary clinical response. In fact, constant relapse was observed after treatment discontinuation.

Interestingly, the first CDI episode occurred in May 2022 after treatment of group A beta-haemolytic *Streptococcus* (SBEGA) infection with amoxicillin. At that time, after failure of metronidazole, the infection was successfully treated with vancomycin (Table 1). However, in May 2023, CDI relapsed after a new episode of SBEGA infection, treated with cefixime. After that, vancomycin was also only temporarily beneficial, as was fidaxomicin, with different regimens (Table 1) and with a persistent CDI outcome. Therefore, the medical staff of Meyer Children’s Hospital IRCCS asked for second opinion and possible FMT at Bambino Gesù Children’s Hospital, IRCCS (OPBG), a referral centre for this treatment in Italy.

**Figure 1:**
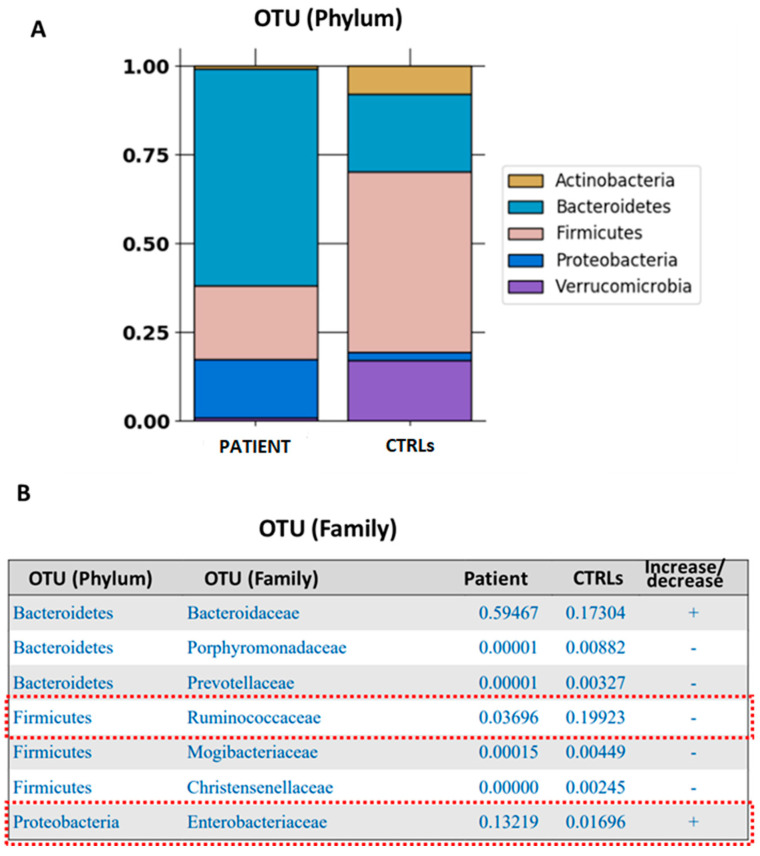
Diagnostic map of the GM of the CFSPID patient compared to the GM profiles of a reference age-matched healthy subject group, exploited as a control (CTRL) group. The histogram refers to OTU distributions at the phylum (**A**) and family (**B**) levels.

The first evaluation at OPBG was in November 2023 after the VI episode of rCDI. The infectious diseases team reviewed the patient’s infectious and pharmacological history. At that time, two weeks after the last fidaxomicin discontinuation, the Microbiomics Unit performed the first GM map by 16S rRNA analysis of three stool samples and produced a GM diagnostic report (Figure 1).

Briefly, using the metataxonomy method for diagnosis of gut dysbiosis (OPBG patent N° WO2017216820A1, https://patents.google.com/patent/WO2017216820A1/en, accessed on 8 October 2024), a degree of gut dysbiosis, expressed as per the microbial dysbiosis index (MDI), was assigned to the CF patient’s GM through comparison of the GM profile to that of healthy age-matched subjects, or controls (CTRLs). The method characterized the GM profiles of the CF patient, describing operational taxonomic units (OTUs) at three taxonomic levels, i.e., phylum, family and genus. The MDI was computed by exploiting the quadratic dissimilarity index, Z = (½ × Σ(f_case_ − f_controls_)^2^)^1/2^ × 100 [25], where f_case_ represents the median value of the OTU distributions at the phylum, family and genus levels of the fecal GM of the CF patient, and f_controls_ describes the median value of OTU distributions at the same taxonomic levels of the fecal GM of CTRLs. A value of MDI = 0 indicates no dissimilarity, while MDI = 1 represents maximum dissimilarity, hence representing a measure of dysbiosis. The MDI was classified as mild (<25%), moderate (25–35%) and high (>35%), accordingly to a study on pediatric IBD [26].

The GM profile of the patient presented a high level of dysbiosis (35%), with a high prevalence at the phylum level of Bacteroidetes and Proteobacteria and a reduction in Firmicutes compared to the healthy age-matched control group (CTRL). Moreover, almost a complete depletion of both Actinobacteria and Verrucomicrobia was observed (Figure 1A). Concerning OTU distributions at the family level, Ruminococcaceae was reduced by 5.5 times, while Enterobacteriaceae increased by 780 times, compared to CTRLs (Figure 1B).

**Figure 2:**
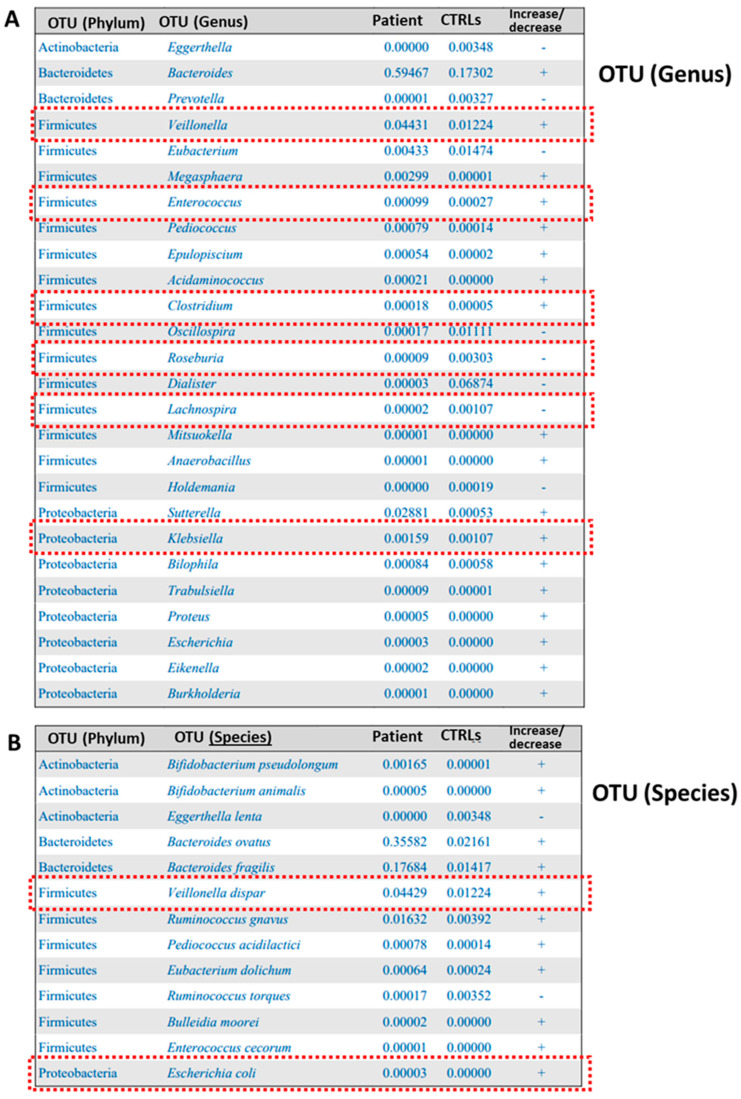
Diagnostic map of the GM of the CFSPID patient compared to the GM profiles of a reference age-matched healthy subject group, exploited as a control (CTRL). The histogram refers to OTU distributions at the genus (**A**) and species (**B**) levels.

At the genus level, an increase in *Veillonella, Enterococcus* and *Clostridium* (Firmicutes) as well as an overabundance of *Sutterella* and *Klebsiella* (Proteobacteria) (Figure 2A) were observed, while at the species level, *Veillonella dispar* and *Escherichia coli* were increased (Figure 2B).

Based on rCDI episodes, absent or limited responsiveness to multiple rounds of antibiotic therapy and evidence of dysbosis, the multidisciplinary team of clinicians and microbiologists from OPBG and the Meyer Children’s Hospital decided to proceed with compassionate use of FMT. The medical history report and treatment proposal for this single case was submitted to the National Transplant Center (Centro Nazionale Trapianti—CNT) and to the OPBG Ethics Committees (Prot. n. 977, 20 December 2023, Rome), finally obtaining authorization to proceed.

**Figure 3:**
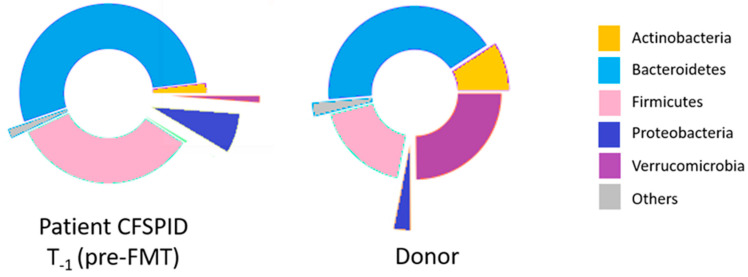
GM map of the CFSPID patient at the pre-FMT T_−1_ time point compared to the GM map of the mother. The cake representations refer to the OTU distributions reported at the phylum level.

In order to identify the best donor for FMT, parents were selected following the FMT screening procedures in use in OPBG. Donor selection was based on anamnestic and clinical interviews, biohumoral and microbiological tests, GM profiles, MDI value and recipient–donor GM map matching (Figure 3) (The gut microbiome precision medicine and the fecal microbiota transplantation in children, Putignani L and OPBG Multidisciplinary Study Group for FMT, Giornale SIGENP, n.1, 2024, https://sigenp.org/giornale-sigenp/, accessed on 8 October 2024).

Following the donor screening results, the mother was considered as an eligible donor (Figure S1).

During FMT authorization and donor selection, the girl experienced her VII (last) rCDI episode. According to the infectious disease unit, another course of fidaxomicin was administered, with plans to continue the therapy until FMT approval.

The child was admitted to the Gastroenterology Unit, asymptomatic, after suspending antibiotic treatment 48 h prior to admission. Stool samples were collected for a new baseline GM analysis immediately before FMT (T_0_). In parallel, a fecal emulsion from the mother’s stool was obtained and frozen, in order to be ready and available for the procedure [19,27].

Then, endoscopic FMT was performed by esophagogastroduodenoscopy (EGD). The post-procedure course was uneventful, and the child was discharged after three days of observation.

**Figure 4:**
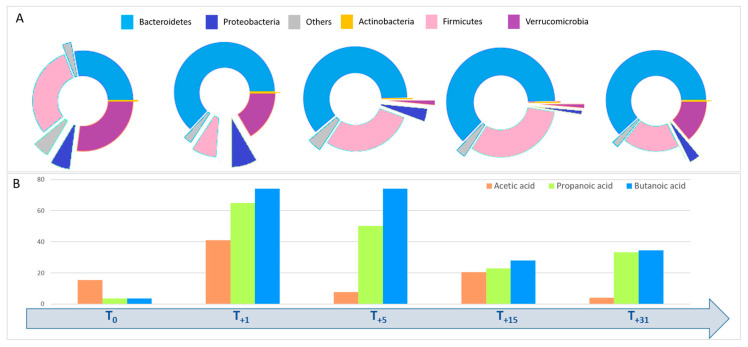
Gut microbiota profiles represented at the phylum level (**A**) and fecal SCFAs levels (**B**) of the patient pre and post FMT at T_0_, T_+1_, T_+5_, T_+15_ and T_+31_ time points, reported as days.

Successful treatment outcomes included both the resolution of rCDI and the restoration of intestinal eubiosis. GM profiling was repeated at different post-FMT time points in order to observe the microbiological “engraftment”: T_+1_ (1 day after transplant), T_+5_, T_+9_, T_+15_ and T_+31_ (Figure 4).

The follow-up analysis after the FMT procedure showed, regarding Actinobacteria, an increase in *Bifidobacterium* and *Collinsella*. Moreover, an increase in Bacteroidetes and a decrease in Firmicutes, in particular *V. dispar*, were observed (Table 2).

Furthermore, to corroborate the amelioration of intestinal dysbiosis after FMT, SCFAs were detected in fecal samples. The concentration of SCFAs was measured by a gas chromatograph (GC) coupled to a mass spectrometer (MS) [28]. Firstly, the patient showed lower levels of SCFAs at the pre-FMT time point compared to the concentrations detected during the time course. In particular, immediately after FMT, higher quantities of acetic, propionic and butyric acids were observed; then, an expected concentration reduction was reported until a constancy of values occurred during follow-up (Table 3), coupled to low level of the dysbiosis index and, especially, to the clearance of gut symptoms.

## 3. Discussion

FMT is a method of changing the GM composition of a subject in order to gain a therapeutic benefit. FMT in children is still used as a potential, experimental therapy to treat or ameliorate different conditions or symptoms. Amongst the different pathological conditions, FMT has been tested in children with IBD and has been shown to be safe and effective in managing symptoms [29,30]. Currently, most clinical experience with FMT has originated from the treatment of recurrent or refractory CD in adulthood. However, it has also been tested in pediatric patients with recurrent *Clostridium difficile* infection [31], with an optimal response in terms of *Clostridioides* eradication. However, the safety and efficacy of using FMT to treat rCDI in children with other pathologies is still largely unexplored.

As observed for other FC-related models characterized by the F508del variant [32], it has been shown that defects in the gene encoding for the cystic fibrosis transmembrane conductance regulator (CFTR) lead to the malfunction of this protein, specifically exerting a block in the transport of chloride and bicarbonate ions and water at the level of the intestinal epithelial tissues [33]. This new GI balance causes, within the GM, a reduction in pH and an anaerobic environment, creating a microenvironment suitable for the growth of microbial species such as *C. difficile*. Normally, commensal bacteria, including Clostridia species such as *Clostridium scindens*, are capable of converting primary bile acids into secondary bile acids in the intestinal lumen, which inhibit the growth of *C. difficile* [34].

However, antibiotic treatment can deplete competing commensal species, including *C. scindens* [35], leading to the accumulation of primary bile acids and an increase in *C. difficile* spore germination [36].

CDI is underestimated in CF because it is typically asymptomatic or displays minimal symptoms and, in any case, there is usually a rapid resolution after vancomycin or metronidazole treatment [37]. Moreover, *C. difficile* in CF patients is not common, and generally diarrhea or other GI symptoms are primarily attributed to reasons different from CDI, such as reduced dosage of pancreatic extracts [38,39]. Surprisingly, in the present case, the *Clostridium* infection developed in absence of CF-related symptoms and after the first antibiotic treatment (amoxicillin) for *Streptococcus* infection. Both ampicillin and amoxicillin have been recognized as potent inducers of *C. difficile* infection [40], and amoxicillin use is usually particularly identified as the highest risk factor for *C. difficile* diarrhea [41].

Alterations in GM composition with signatures of CDI were reported for CF children, including lower species richness and microbial diversity, with a predominance of Firmicutes [42] and Proteobacteria [43], compared to healthy individuals. In particular, *Escherichia* and *Streptococcus* were associated with CDI, while a reduction in microbial taxa linked to gut protection, such as Porphyromonadaceae [44], Ruminococcaceae and Lachnospiraceae, well known as butyric acid producers [45], was reported. Furthermore, increases in *Enterococcus*, *Veillonella*, *Lactobacillus* and Gammaproteobacteria have been reported in the literature [45]. In particular, in rCDIs, a statistically significant increase in *Veillonella*, Enterobacteriaceae, Streptococci and *Parabacteroides* [46] was observed, consistent with the findings presented in this case report.

The prevalence of *Enterococcus* and *Veillonella* is also a specific signature in CF patients, as is the depletion of *Roseburia* and *Ruminococcus*, as reported in the literature [32]. Moreover, a large increase in *Eubacterium dolichum* and a depletion of *Bifidobacterium longum* are typically observed in CF patients’ GM profile [47].

Moreover, beyond GM dysbiosis, a significant reduction in gut microbial metabolites, such as secondary bile acids and SCFAs, are also observed in CDI patients [48]. In particular, a decrease in butyric acid-producing bacteria such as Ruminococcaceae, Lachnospiraceae and *Clostridium* cluster IV e XIVa has been reported [45]. In this case, a decrease in Ruminococcaceae and Lachnospira and metabolites, such as butyrric acid during CD colonization, was also observed, consistent with the literature.

In order to obtain the best possible engraftment of transplanted GM, a donor with ideal characteristics was selected. In this case, the GM profile of the donor showed a beneficial prevalence of Bacteroidetes and Actinobacteria, with particular reference to *Bifidobacterium longum* (relative abundance of 1.318)*,* which has been thoroughly described to have positive effects for CDI [49]. Moreover, the donor presented a specific strain of *Clostridium hiranonis*, which has been reported to impact the metabolism, growth and toxin production of *C. difficile* [50].

After FMT, the recovery of Bacteroidetes and a decrease in *V. dispar* [51] were observed, confirming the regression of the CDI and the restoration of GM eubiosis.

Finally, a restoration of metabolic and ecological activities was observed after FMT. In particular, in the patient, the regression of the CDI was associated with an increase in Ruminococcaceae and *Collinsella*, producers of butyrate and bile salts, metabolites that typically decrease in CDI, as also observed for pediatric FMT used to treat pediatric IBD [52]. Additionally, SCFAs and secondary bile acid production may increase resistance against CDI by improving the intestinal barrier, the anti-inflammatory response and through the direct inhibition of *C. difficile* [53].

From an ecological point of view, at T_+1_, an increase in facultative anaerobes, including Proteobacteria, was observed, which could have been a transient result of the O_2_ conditions created during the FMT procedures. Afterward, the gradual increase in only anaerobes from the donor (e.g., Bacteroidetes, Firmicutes and Verrucomicrobia) reduced the O_2_ conditions, suppressing the relative amount of Proteobacteria. Interestingly, Actinobacteria maintained low concentrations, probably due to their low kinetics of growth. A high growth rate of SCFA-producing microbes was also confirmed. In fact, compared to other the time points, we showed a peak increase in all SCFAs at T_+1_ and a decrement at T_+5_ with a stable, beneficial concentration level during the entire following time course. The high rate of beneficial bacteria after the FMT procedure could be explained by the donor’s GM composition, with a high presence of Ruminococcaceae and *B. longum*, both SCFA producers [54].

## 4. Conclusions

FMT is considered a standard treatment for the resolution of recurrent *C. difficile* infection in adults, compared to alternative treatments such as antibiotic treatment. Today, FMT is still an experimental treatment in children; hence, more studies are required to assess its safety and efficacy. However, for the current case, FMT actually represented an eradication solution for rCDI treatment in CFSPID, generating CD clearance and GM wellness, as reported by the restoration of intestinal eubiosis and the increase in SCFAs. In the future, the use of personalized FMT for different pediatric patients and conditions, including CF, is expected to act as a clinical treatment.

## Figures and Tables

**Table 1:** rCDI episodes and antibiotic therapies exploited during the period May 2022 to November 2023 for the CFSPID patient.

rCDI Episode	Antibiotic Therapy (Type, Dosage, Administration Time and Route)
I: May 2022	metronidazole 125 mg TID * (10 days), oral administration
II: July 2022	vancomycin 125 mg QID * (10 days), oral administration
III: May 2023	vancomycin 125 mg QID * (10 days), oral administration
IV: July 2023	vancomycin 125 mg QID * followed by tapered regimen (total 45 days), oral administration
V: September 2023	fidaxomicin 200 mg BID * (10 days), oral administration
VI: October 2023	fidaxomicin 200 mg BID * followed by pulsed regimen (total 25 days), oral administration
VII: November 2023	fidaxomicin 200 mg BID * followed by pulsed regimen (total 25 days), oral administration

* BID, bis in die; TIQ, ter in die; QID, quater in die.

**Table 2:** Relative abundance of the main GM bacterial taxa of the CFSPID patient pre and post FMT at T_−1_, T_0_, T_+1_, T_+5_, T_+15_ and T_+31_ time points.

Patient (Recipient)	T_−1_	T_0_	T_+1_	T_+5_	T_+15_	T_+31_	Engraftment
*Enterococcus*	0.090	0.000	0.419	0.004	0.000	0.018	↓
*Clostridium* spp.	0.018	0.007	0.000	0.519	0.040	0.000	↓
*Roseburia*	0.009	0.016	0.019	1.023	0.548	2.243	↑
*Lachnospira*	0.002	0.001	0.000	0.043	0.136	0.050	↑
*Klebsiella*	0.159	0.000	0.100	0.000	0.000	0.000	↓
*Escherichia coli*	0.003	5.07	0.000	0.000	0.000	0.001	↓
*Veillonella dispar*	4.429	0.000	2.997	0.002	0.001	0.594	↓
*Collinsella*	0.000	0.000	0.008	0.146	0.105	0.031	↑
*Bifidobacterium longum*	0.000	0.003	0.047	0.033	0.036	0.028	↑

**Table 3:** Concentration of fecal SCFAs of the CFSPID patient pre and post FMT at T_−1_, T_0_, T_+1_, T_+5_, T_+15_ and T_+31_ time points, reported as days.

Concentration of Main SCFAs (mg/kg)			
Time Points	Acetic Acid	Propionic Acid	Butyric Acid
T_−1_	21.74 ± 15.39	24.64 ± 1.70	8.65 ± 4.06
T_0_	43.80 ± 15.37	60.40 ± 3.53	41.20 ± 3.65
T_1_	88.27 ± 40.94	113.70 ± 64.82	128.50 ± 74.05
T_5_	22.14 ± 7.66	67.97 ± 50.17	105.22 ± 74.21
T_15_	17.99 ± 20.53	34.51 ± 23.02	47.14 ± 28.06
T_31_	14.86 ± 4.00	36.92 ± 33.19	58.26 ± 34.48

## Data Availability

The raw data supporting the conclusions of this article will be made available by the authors on request.

## Supplementary Materials

The following supporting information can be downloaded at: https://www.mdpi.com/article/10.3390/microorganisms12102059/s1, Figure S1: Diagnostic map of the GM of the donor compared to the GM profiles of a reference age-matched healthy subject group, exploited as a control (CTRL) group. Table S1: List of publications on FTM in children published between 2014 and 2024.
