# Supplementary material for: Fecal Microbiota Transplantation for Recurrent Clostridioides difficile Infections in a Cystic Fibrosis Child Previously Screen Positive, Inconclusive Diagnosis (CFSPID): A Case Report
Source: Microorganisms. 2024 Oct 12;12(10):2059. doi: 10.3390/microorganisms12102059 (PMC11509880; doi:10.3390/microorganisms12102059)
Supplement: Supplementary file 1 [file microorganisms-12-02059-s001.zip › microorganisms-3252727-supplementary.pdf]

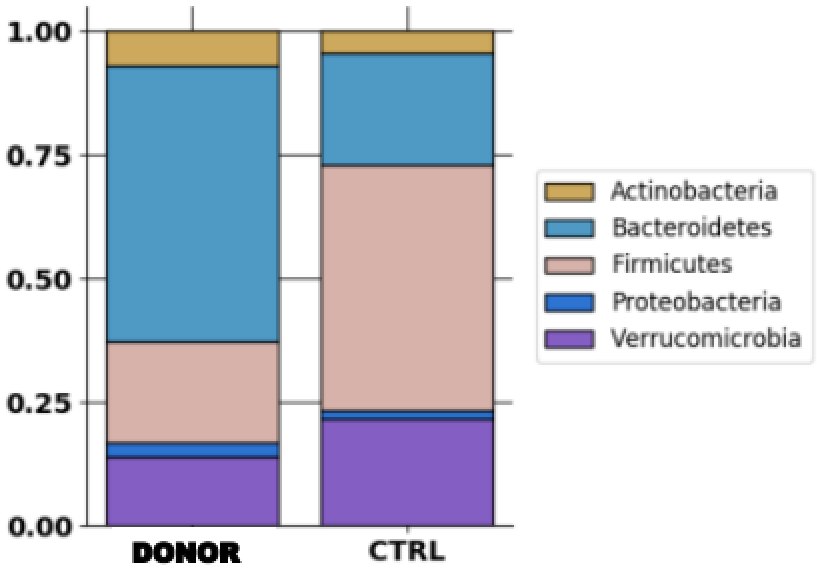

Figure S1: Diagnostic map of the GM of the donor compared to the GM profiles of a reference age-matched healthy subject group, exploited as control (CTRL) group.

Table S1. List of principal publications on FTM in children published between 2014 and 2024[a]

Authors	Title	Journal/Book	Publication Year	DOI or PUBLICATION LINK
Youngster I. et al.	Oral, capsulized, frozen fecal microbiota transplantation for relapsing Clostridium difficile infection	JAMA	2014	10.1001/jama.2014.13875
Walia R. et al.	Efficacy of fecal microbiota transplantation in 2 children with recurrent Clostridium difficile infection and its impact on their growth and gut microbiome	J Pediatr Gastroenterol Nutr	2014	10.1097/MPG.0000000000000495
Kelly CR. et al.	Fecal microbiota transplant for treatment of Clostridium difficile infection in immunocompromised patients	Am J Gastroenterol	2014	10.1038/ajg.2014.133
Russell GH. et al.	Fecal transplant for recurrent Clostridium difficile infection in children with and without inflammatory bowel disease	J Pediatr Gastroenterol Nutr	2014	10.1097/MPG.0000000000000283
Wei Y. et al.	Fecal microbiota transplantation restores dysbiosis in patients with methicillin resistant Staphylococcus aureus enterocolitis	BMC Infect Dis	2015	10.1186/s12879-015-0973-1
Paramsothy S. et al.	Donor Recruitment for Fecal Microbiota Transplantation	Inflamm Bowel Dis	2015	10.1097/MIB.0000000000000405
Suskind DL. et al.	Fecal microbial transplant via nasogastric tube for active pediatric ulcerative colitis	J Pediatr Gastroenterol Nutr	2015	10.1097/MPG.0000000000000544
Wei Y. et al.	Pectin enhances the effect of fecal microbiota transplantation in ulcerative colitis by delaying the loss of diversity of gut flora	BMC Microbiol	2016	10.1186/s12866-016-0869-2
Brittnacher MJ. et al.	GUTSS: An Alignment-Free Sequence Comparison Method for Use in Human Intestinal Microbiome and Fecal Microbiota Transplantation Analysis	PLoS One	2016	10.1371/journal.pone.0158897
Shimizu H. et al.	Repeated fecal microbiota transplantation in a child with ulcerative colitis	Pediatr Int	2016	10.1111/ped.12967
Liu SX. et al.	Fecal microbiota transplantation induces remission of infantile allergic colitis through gut microbiota re-establishment	World J Gastroenterol	2017	10.3748/wjg.v23.i48.8570
Lahtinen P. et al.	Faecal microbiota transplantation in patients with Clostridium difficile and significant comorbidities as well as in patients with new indications: A case series	World J Gastroenterol	2017	10.3748/wjg.v23.i39.7174
Pai N. et al.	Protocol for a randomised, placebo-controlled pilot study for assessing feasibility and efficacy of faecal microbiota transplantation in a paediatric ulcerative colitis population: PediFETCh trial	BMJ Open	2017	10.1136/bmjopen-2017-016698

He Z. et al.	Multiple fresh fecal microbiota transplants induces and maintains clinical remission in Crohn's disease complicated with inflammatory mass	Sci Rep	2017	10.1038/s41598-017-04984-z
Kang DW. et al.	Microbiota Transfer Therapy alters gut ecosystem and improves gastrointestinal and autism symptoms: an open-label study	Microbiome	2017	10.1186/s40168-016-0225-7
Suez J. et al.	Post-Antibiotic Gut Mucosal Microbiome Reconstitution Is Impaired by Probiotics and Improved by Autologous FMT	Cell	2018	10.1016/j.cell.2018.08.047
Barfuss S. et al.	Cardiac allograft vasculopathy following fecal microbiota transplantation for recurrent C. difficile infection	Transpl Infect Dis	2018	10.1111/tid.12983
Halkjær SI. et al.	Faecal microbiota transplantation alters gut microbiota in patients with irritable bowel syndrome: results from a randomised, double-blind placebo-controlled study	Gut	2018	10.1136/gutjnl-2018-316434
Bulik-Sullivan EC. et al.	Intestinal Microbial and Metabolic Alterations Following Successful Fecal Microbiota Transplant for D-Lactic Acidosis	J Pediatr Gastroenterol Nutr	2018	10.1097/MPG.0000000000002043
Dinh A. et al.	Clearance of carbapenem-resistant Enterobacteriaceae vs vancomycin-resistant enterococci carriage after faecal microbiota transplant: a prospective comparative study	J Hosp Infect	2018	10.1016/j.jhin.2018.02.018
Garza-González E. et al.	Intestinal Microbiome Changes in Fecal Microbiota Transplant (FMT) vs. FMT Enriched with Lactobacillus in the Treatment of Recurrent Clostridioides difficile Infection	Can J Gastroenterol Hepatol	2019	10.1155/2019/4549298
Pai N. et al.	Protocol for a double-blind, randomised, placebo-controlled pilot study for assessing the feasibility and efficacy of faecal microbiota transplant in a paediatric Crohn's disease population: PediCRaFT Trial	BMJ Open	2019	10.1136/bmjopen-2019-030120
Quraishi MNN. et al.	STOP-Colitis pilot trial protocol: a prospective, open-label, randomised pilot study to assess two possible routes of faecal microbiota transplant delivery in patients with ulcerative colitis	BMJ Open	2019	10.1136/bmjopen-2019-030659
Zhong S. et al.	Fecal microbiota transplantation for refractory diarrhea in immunocompromised diseases: a pediatric case report	Ital J Pediatr	2019	10.1186/s13052-019-0708-9
Kang DW. et al.	Long-term benefit of Microbiota Transfer Therapy on autism symptoms and gut microbiota	Sci Rep	2019	10.1038/s41598-019-42183-0

Moutinho BD. et al.	Fecal microbiota transplantation in refractory ulcerative colitis - a case report	J Int Med Res	2019	10.1177/0300060518821790
Aldrich AM. et al.	Analysis of Treatment Outcomes for Recurrent Clostridium difficile Infections and Fecal Microbiota Transplantation in a Pediatric Hospital	Pediatr Infect Dis J	2019	10.1097/INF.0000000000002053
Leong KSW. et al.	Effects of Fecal Microbiome Transfer in Adolescents With Obesity: The Gut Bugs Randomized Controlled Trial	JAMA Netw Open	2020	10.1001/jamanetworkopen.2020.30415
Kang DW. et al.	Distinct Fecal and Plasma Metabolites in Children with Autism Spectrum Disorders and Their Modulation after Microbiota Transfer Therapy	mSphere	2020	10.1128/mSphere.00314-20
Li Q. et al.	Fecal Microbiota Transplantation for Ulcerative Colitis: The Optimum Timing and Gut Microbiota as Predictors for Long-Term Clinical Outcomes	Clin Transl Gastroenterol	2020	10.14309/ctg.0000000000000224
Wasinger VC. et al.	Spp24 is associated with endocytic signalling, lipid metabolism, and discrimination of tissue integrity for 'leaky-gut' in inflammatory bowel disease	Sci Rep	2020	10.1038/s41598-020-69746-w
Wang Y. et al.	Pilot study of cytokine changes evaluation after fecal microbiota transplantation in patients with ulcerative colitis	Int Immunopharmacol	2020	10.1016/j.intimp.2020.106661
Lahtinen P. et al.	Randomised clinical trial: faecal microbiota transplantation versus autologous placebo administered via colonoscopy in irritable bowel syndrome	Aliment Pharmacol Ther	2020	10.1111/apt.15740
Spinner JA. et al.	Fecal microbiota transplantation in a toddler after heart transplant was a safe and effective treatment for recurrent Clostridiodes difficile infection: A case report	Pediatr Transplant	2020	10.1111/petr.13598
Merli P. et al.	Decolonization of multi-drug resistant bacteria by fecal microbiota transplantation in five pediatric patients before allogeneic hematopoietic stem cell transplantation: gut microbiota profiling, infectious and clinical outcomes	Haematologica	2020	10.3324/haematol.2019.244210
Quagliariello A. et al.	Fecal Microbiota Transplant in Two Ulcerative Colitis Pediatric Cases: Gut Microbiota and Clinical Course Correlations	Microorganisms	2020	10.3390/microorganisms8101486
Lai J. et al.	New Evidence of Gut Microbiota Involvement in the Neuropathogenesis of Bipolar Depression by TRANK1 Modulation: Joint Clinical and Animal Data	Front Immunol	2021	10.3389/fimmu.2021.789647

Zhao Y. et al.	Safety and Efficacy of Fecal Microbiota Transplantation for Grade IV Steroid Refractory GI-GvHD Patients: Interim Results From FMT2017002 Trial	Front Immunol	2021	10.3389/fimmu.2021.678476
Wilson BC. et al.	Strain engraftment competition and functional augmentation in a multi-donor fecal microbiota transplantation trial for obesity	Microbiome	2021	10.1186/s40168-021-01060-7
Clancy AK. et al.	Dietary Intakes of Recipients of Faecal Microbiota Transplantation: An Observational Pilot Study	Nutrients	2021	10.3390/nu13051487
Karolewska-Bochenek K. et al.	Faecal Microbiota Transfer - a new concept for treating cytomegalovirus colitis in children with ulcerative colitis	Ann Agric Environ Med	2021	10.26444/aaem/118189
Wu W. et al.	Fecal microbiota transplantation before hematopoietic stem cell transplantation in a pediatric case of chronic diarrhea with a FOXP3 mutation	Pediatr Neonatol	2021	10.1016/j.pedneo.2020.11.003
Sabus A. et al.	Fecal Microbiota Transplantation for Treatment of Severe Clostridioides difficile Colitis in a Pediatric Patient With Non-Hodgkin Lymphoma	J Pediatr Hematol Oncol	2021	10.1097/MPH.0000000000002023
Popov J. et al.	Pediatric Patient and Parent Perceptions of Fecal Microbiota Transplantation for the Treatment of Ulcerative Colitis	J Pediatr Gastroenterol Nutr	2021	10.1097/MPG.0000000000002995
de Groot P. et al.	Faecal microbiota transplantation halts progression of human new-onset type 1 diabetes in a randomised controlled trial	Gut	2021	10.1136/gutjnl-2020-322630
Kelly CR. et al.	Fecal Microbiota Transplantation Is Highly Effective in Real-World Practice: Initial Results From the FMT National Registry	Gastroenterology	2021	10.1053/j.gastro.2020.09.038
Holvoet T. et al.	Fecal Microbiota Transplantation Reduces Symptoms in Some Patients With Irritable Bowel Syndrome With Predominant Abdominal Bloating: Short- and Long-term Results From a Placebo-Controlled Randomized Trial	Gastroenterology	2021	10.1053/j.gastro.2020.07.013
Wei S. et al.	Cross-generational bacterial strain transfer to an infant after fecal microbiota transplantation to a pregnant patient: a case report	Microbiome	2022	10.1186/s40168-022-01394-w
Carpén N. et al.	Transplantation of maternal intestinal flora to the newborn after elective cesarean section (SECFLOR): study protocol for a double blinded randomized controlled trial	BMC Pediatr	2022	10.1186/s12887-022-03609-3

Ye C. et al.	[Establishment and preliminary clinical application of human intestinal fluid transplantation]	Zhonghua Wei Chang Wai Ke Za Zhi	2022	10.3760/cma.j.cn441530-20220601-00239
Li X. et al.	Characteristics and management of children with Clostridioides difficile infection at a tertiary pediatric hospital in China	Braz J Infect Dis	2022	10.1016/j.bjid.2022.102380
Tamura S. et al.	[Fecal microbiota transplantation for refractory Clostridioides difficile infection with Crohn's disease in an allogeneic bone marrow transplant recipient]	Rinsho Ketsueki	2022	10.11406/rinketsu.63.217
Chen Y. et al.	FTACMT study protocol: a multicentre, double-blind, randomised, placebo-controlled trial of faecal microbiota transplantation for autism spectrum disorder	BMJ Open	2022	10.1136/bmjopen-2021-051613
Mendelsohn RB. et al.	Fecal Microbiota Transplantation Is Safe for Clostridiodies difficile Infection in Patients with Solid Tumors Undergoing Chemotherapy	Dig Dis Sci	2022	10.1007/s10620-021-07024-z
Merli P. et al.	Fecal microbiota transplantation for the treatment of steroid-refractory, intestinal, graft-versus-host disease in a pediatric patient	Bone Marrow Transplant	2022	10.1038/s41409-022-01752-0
Ianiro et al.,	Variability of strain engraftment and predictability of microbiome composition after fecal microbiota transplantation across different diseases.	Nat Med	2022	10.1038/s41591-022-01964-3
Piazzesi A. et al.	Case Report: The impact of severe cryptosporidiosis on the gut microbiota of a pediatric patient with CD40L immunodeficiency	Front Cell Infect Microbiol	2023	10.3389/fcimb.2023.1281440
Conover KR. et al.	Fecal Microbiota Transplantation for Clostridioides difficile Infection in Immunocompromised Pediatric Patients	J Pediatr Gastroenterol Nutr	2023	10.1097/MPG.0000000000003714
Liu T. et al.	Treatment of Radiation Enteritis With Fecal Transplantation	Am Surg	2023	10.1177/00031348221091954
Bracaglia et al.	Microbiota transplant to control inflammation in a patient with NLRC4 gain-of-function–induced disease	American Academy of Allergy, Asthma and Immunology	2023	doi.org/10.1016/j.jaci.2023.03.031
Zhang YJ. et al.	Higher alpha diversity and Lactobacillus blooms are associated with better engraftment after fecal microbiota transplant in inflammatory bowel disease	Sci Rep	2024	10.1038/s41598-024-68619-w
Zuppi M. et al.	Fecal microbiota transplantation alters gut phage communities in a clinical trial for obesity	Microbiome	2024	10.1186/s40168-024-01833-w

Tweedie-Cullen RY. et al.	Protocol for the Gut Bugs in Autism Trial: a double-blind randomised placebo-controlled trial of faecal microbiome transfer for the treatment of gastrointestinal symptoms in autistic adolescents and adults	BMJ Open	2024	10.1136/bmjopen-2023-074625
Putignani L and OPBG Multidisciplinary Study Group for FMT	The gut microbiome precision medicine and the faecal microbiota transplantation in children,	SIGENP Journal	2024	https://sigenp.org/giornale-sigenp/

[a]The reported publications have been selected consistently with: clinical trials; observational studies; multicentre study; in humans; in child 0-18 years old.
